# Development of a Stability-Indicating Stereoselective Method for Quantification of the Enantiomer in the Drug Substance and Pharmaceutical Dosage Form of Rosuvastatin Calcium by an Enhanced Approach

**DOI:** 10.3797/scipharm.1410-09

**Published:** 2014-12-05

**Authors:** Gangireddy Rajendra Reddy, Papammagari Ravindra Reddy, Polisetty Siva Jyothi

**Affiliations:** Department of Chemistry, Sri Krishnadevaraya University, Anantapur 515001, A.P, India

**Keywords:** Stereoselective method, Stability-indicating method, ICH guidelines, Forced degradation, Immobilized cellulose stationary phase (Chiralpak IB)

## Abstract

A novel, simple, precise, and stability-indicating stereoselective method was developed and validated for the accurate quantification of the enantiomer in the drug substance and pharmaceutical dosage forms of Rosuvastatin Calcium. The method is capable of quantifying the enantiomer in the presence of other related substances. The chromatographic separation was achieved with an immobilized cellulose stationary phase (Chiralpak IB) 250 mm x 4.6 mm x 5.0 μm particle size column with a mobile phase containing a mixture of n-hexane, dichloromethane, 2-propanol, and trifluoroacetic acid in the ratio 82:10:8:0.2 (v/v/v/v). The eluted compounds were monitored at 243 nm and the run time was 18 min. Multivariate analysis and statistical tools were used to develop this highly robust method in a short span of time. The stability-indicating power of the method was established by subjecting Rosuvastatin Calcium to the stress conditions (forced degradation) of acid, base, oxidative, thermal, humidity, and photolytic degradation. Major degradation products were identified and found to be well-resolved from the enantiomer peak, proving the stability-indicating power of the method. The developed method was validated as per International Conference on Harmonization (ICH) guidelines with respect to specificity, limit of detection and limit of quantification, precision, linearity, accuracy, and robustness. The method exhibited consistent, high-quality recoveries (100 ± 10%) with a high precision for the enantiomer. Linear regression analysis revealed an excellent correlation between the peak responses and concentrations (r^2^ value of 0.9977) for the enantiomer. The method is sensitive enough to quantify the enantiomer above 0.04% and detect the enantiomer above 0.015% in Rosuvastatin Calcium. The stability tests were also performed on the drug substances as per ICH norms.

## Introduction

Rosuvastatin Calcium is classified as a statin, a type of agent that inhibits cholesterol production in the liver. Rosuvastatin Calcium is a synthetic lipid-lowering agent approved as a treatment for hypercholesterolemia. Rosuvastatin Calcium is a selective and competitive inhibitor of 3-hydroxy-3-methylglutaryl coenzyme A (HMG-CoA) reductase, the rate-limiting enzyme that converts HMG-CoA to mevalonate, an early and rate-limiting step in cholesterol biosynthesis. The primary site of action of Rosuvastatin is the liver, the target organ for cholesterol lowering. Rosuvastatin reduces cholesterol by increasing the number of low-density lipoprotein (LDL) receptors on the cell surface to enhance uptake and catabolism of LDL. It also inhibits hepatic synthesis of hepatic very-low-density lipoprotein (VLDL), which reduces the total number of VLDL and LDL particles. The treatment reduces triglycerides (TG) and produces increases in high-density lipoprotein cholesterol (HDL-C) [1–6].

Stereoselectivity plays a major role in the drug’s action and single enantiomer drugs are safer, better-tolerated, and more efficient [7, 8]. According to our knowledge, there is no stereoselective HPLC method for the quantification of the enantiomer at the 0.15% level existing in the literature of Rosuvastatin Calcium. The reported analytical methods that relate to Rosuvastatin Calcium are three mass detection methods for the determination of Rosuvastatin Calcium in plasma and biological fluids [9–11], a stability-indicating related substances method by UPLC [12], two stability-indicating HPLC methods [13, 14], one HPTLC method [14], and three UV spectrophotometric methods [14–16] for the quantification of Rosuvastatin Calcium in tablets. But none of the methods either individually or combined are capable of quantifying the enantiomer of Rosuvastatin Calcium.

In this paper, we describe the development of a stability-indicating stereoselective method for the quantification of the enantiomer in the drug substance and pharmaceutical dosage forms of Rosuvastatin Calcium. ICH Q11 guidelines describe enhanced approaches for manufacturing processes [17]. We have applied the same principles to analytical method development. Selecting the column stationary phase on the basis of functional groups present in the compound, applying the understanding gained from initial screening for the selection of factors for the design of experiments (DoE), finalizing the method’s conditions on the basis of a thorough understanding gained from multivariate analysis about the edges of failure (design space), and the inclusion of comparatively green solvents [18] wherever possible are considered as an enhanced approach here.

## Experimental

### Standards, Chemicals, and Reagents

**Fig. 1:**
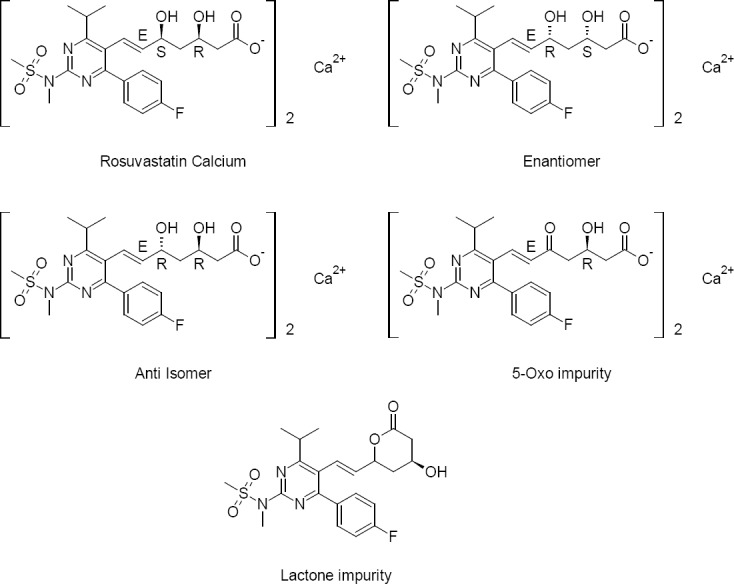
Structures of Rosuvastatin Calcium, the enantiomer, and its possible known degradation impurities

Rosuvastatin Calcium is available as tablets with the brand name Crestor with a label claim of 5, 10, 20, and 40 mg of the drug. The HPLC grade n-hexane, 2-propanol (IPA), dichloromethane (DCM), and methanol were procured from Rankem, India. Trifluoroacetic acid was procured from Acros, Belgium. Structures of Rosuvastatin Calcium, the enantiomer, and its possible known degradation impurities are shown in Figure 1.

### Instruments and Software

#### HPLC1

The Waters Alliance 2695 Separation Module equipped with PDA (Waters Corporation, Milford, MA, USA) was used for development studies and specificity.

#### HPLC2

The Agilent 1200 Series equipped with VWD (Agilent Technologies, Waldron, Germany) was used for validation parameters.

HPLC1 and HPLC2 were monitored with Empower 2 software (Waters Corporation, Milford, MA, USA).

A calibrated electronic single pan balance (Mettler Toledo, Greifensee, Switzerland) and Ultrasonicator (Bandelin Sonorex, Berlin, Germany) were also used during the analysis. A digital water bath (Cintex, Mumbai, India) was used for the hydrolysis studies. Photostability studies were carried out in a photostability chamber (Sanyo, Leicestershire, UK). Thermal stability studies were performed in a dry air oven (Cintex, Mumbai, India).

Design-Expert version 9.0.1 (Stat-Ease Inc., Minneapolis) was used for central composite design construction and interpretation. Microsoft Excel 2007 was used for the analysis of validation results.

### Chromatographic Conditions

The method was developed using a Chiralpak IB 250 mm x 4.6 mm x 5.0 μm particle size column (Diacel Chiral Technologies) with a mobile phase containing a mixture of n-hexane, dichloromethane, 2-propanol, and trifluoroacetic acid in the ratio 82:10:8:0.2 (v/v/v/v). The flow rate of the mobile phase was 1.0 mL min^−1^ (isocratic). The column oven temperature was maintained at 25°C and the sampler cooler was maintained at 10°C. The chromatogram was monitored at 243 nm wavelength. The injection volume was 10 μL. Dichloromethane and methanol in the ratio 96:4 (v/v) were used as the diluent for all preparations. The run time was 18 minutes. The sample concentration was 1000 ppm (1.0 mg mL^−1^).

### Stock Solution Preparation (30 ppm)

Fifteen mg of the Rosuvastatin Calcium enantiomer standard was dissolved in 2 mL of methanol and made up to 25 mL with diluent. Then 2.5 mL of the above stock solution was diluted to 50 mL with diluent.

### System Suitability Solution Preparation (Rosuvastatin Calcium 1000 ppm, Enantiomer 3 ppm)

Fifty mg of Rosuvastatin Calcium standard was dissolved in 2 mL of methanol and 5 mL of the above stock solution was added and made up to 50 mL with diluent.

### Standard Solution Preparation (1.5 ppm)

An amount of 2.5 mL of the above stock solution was diluted to 50 mL with diluent.

### Tablet Analysis

Two Crestor tablets (each tablet contains 40 mg of Rosuvastatin Calcium) were crushed into a fine powder; an amount equivalent to 50 mg Rosuvastatin Calcium was dissolved in 2 mL of methanol, made up to 50 mL with diluent, and the filtered solution was injected (excipients were microcrystalline cellulose, lactone monohydrate, tribasic calcium phosphate, crospovidone, magnesium stearate, hypromellose, triacetin, titanium dioxide, yellow, and red ferric oxide).

### Blend Solution Preparation

For accurate quantification, it is necessary to ensure the separation of other related substances from the enantiomer. Hence, a blend solution containing 1.0 mg mL^−1^ solution of Rosuvastatin Calcium and a 0.15% level of the enantiomer and all impurities was prepared (blend solution-1) and used for method development studies.

A blend solution (blend solution-2) containing 1.0 mg mL^−1^ solution of Rosuvastatin Calcium and a 0.15% level of the enantiomer and anti isomer (the anti isomer was included to ensure its separation from the enantiomer as it was the close-eluting impurity) was prepared and used for DoE.

## Method Validation

The method has been validated as per ICH guidelines Q2 (R1). The method was validated for the following parameters: system suitability, specificity/forced degradation studies, limit of quantitation (LOQ) and limit of detection (LOD), precision, linearity, accuracy, robustness, solution stability, and mobile phase stability [19].

### Specificity/Forced Degradation Studies

Specificity is the ability of the method to measure the analyte response in the presence of its potential impurities. The specificity of the developed HPLC method was carried out in the presence of the enantiomer. Specificity was demonstrated by spiking the test Rosuvastatin Calcium concentration with 0.15% of the enantiomer. An equivalent placebo concentration was prepared and injected into the chiral system to evaluate the interference with the enantiomer. Forced degradation studies were carried out at an initial concentration of 1.0 mg mL^−1^ of Rosuvastatin Calcium to provide an indication of the stability-indicating property and specificity of the proposed method [20, 21]. For this stereoselective method, it is necessary to ensure that no degradation product, which may form under various stress conditions, interferes with the enantiomer.

Intentional degradation was attempted for the following stress conditions:

**Table:**
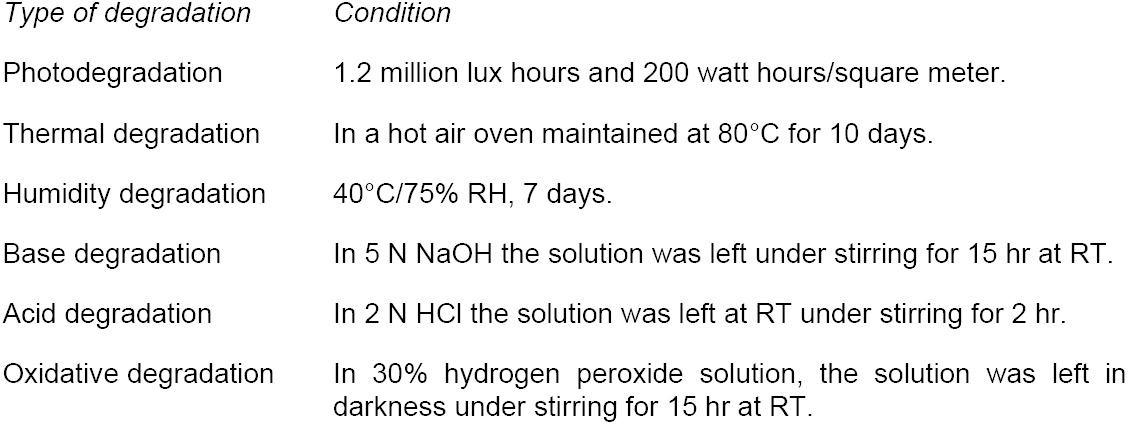


#### Limit of Detection and Quantification

The limit of detection (LOD) and limit of quantification (LOQ) for the enantiomer was estimated at signal-to-noise ratios of 3:1 and 10:1, respectively, by injecting a series of diluted solutions with known concentrations. A precision study was also conducted at the LOQ level by injecting six individual preparations of the enantiomer and calculating the % relative standard deviation (% RSD) of the area. The accuracy at the LOQ level was evaluated in triplicate for the enantiomer by spiking the impurity at the estimated LOQ level to the test solution.

#### Precision

The repeatability of the impurities method was verified by injecting six individual preparations. Rosuvastatin Calcium was spiked with 0.15% of the enantiomer with respect to the test concentration (1000 ppm) and the %RSD was calculated for enantiomer content. The intermediate precision of the method was also determined by repeating the same experiment on different days by different analysts using different equipment.

#### Linearity

Linearity solutions for the method of impurities were prepared by diluting impurity stock solutions to the required concentrations. The solutions were prepared at different concentration levels from the limit of quantification (LOQ) 0.4 ppm to 3.0 ppm.

#### Accuracy

Recovery experiments were conducted to determine the accuracy of the impurities method for the quantification of the enantiomer in Rosuvastatin Calcium. The study was conducted by spiking the placebo-based solution of the test sample (1000 ppm) with a known amount of the enantiomer (0.75, 1.50, and 2.25 ppm) in triplicate.

#### Robustness

The robustness of the developed method was evaluated from DoE experiments data and the effects graphs. The effect of flow rate was studied at 0.90 and 1.1 mL min^−1^ and compared with the flow rate of the method at 1.0 mL min^−1^.

## Results and Discussion

### Method Development and Optimization of Chromatographic Conditions

#### Selection of Detector and Basis for Initial Wavelength Selection

**Fig. 2:**
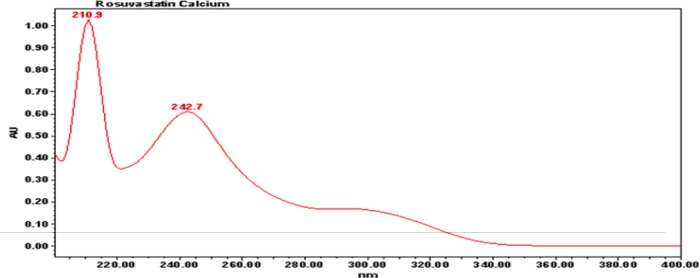
UV spectrum for Rosuvastatin Calcium

Rosuvastatin Calcium solution was prepared in methanol at a concentration of 100 ppm and scanned in an ultraviolet (UV)-visible spectrometer; Rosuvastatin Calcium had maximum UV absorbances at 210 nm and 243 nm (Figure 2). Selection of a wavelength in chiral method development is based on the cutoff values of the solvents used. Hence, detection at 243 nm was selected for the method development process, as the choice of solvents at 243 nm is greater when compared to 210 nm.

**Fig. 3:**
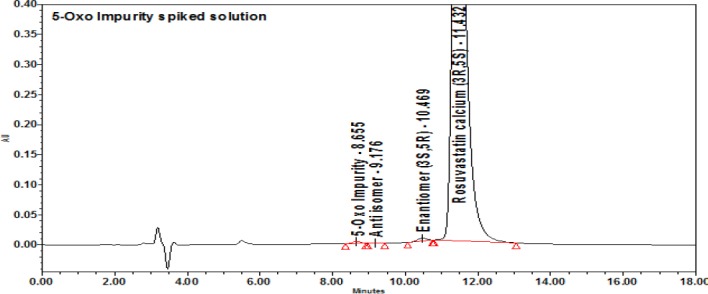
5-Oxo impurity, anti isomer, and enantiomer-spiked chromatogram

**Fig. 4:**
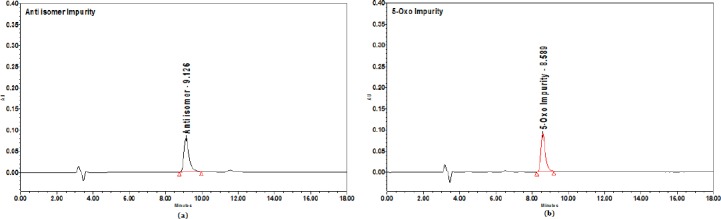
(a) Anti isomer and (b) 5-Oxo impurity chromatograms

**Fig. 5:**
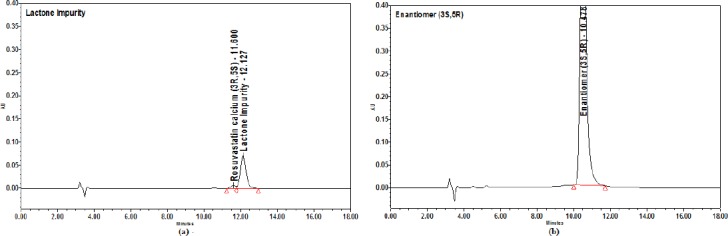
(a) Lactone impurity and (b) enantiomer chromatograms

#### Selection of Column

One of the best chiral stationary phases (CSP) for the separation of aromatic compounds with functional groups such as carbonyl, alcohol, and amine (like Rosuvastatin Calcium) are amylose- and cellulose-based polysaccharide stationary phases [22]. Immobilized polysaccharide columns are compatible with a wide range of solvents and have increased the range of applications (chiral recognition mechanisms) [23]. Hence, the immobilized cellulose-based polysaccharide column Chiralpak IB was selected for screening.

The CSP in Chiralpak IB is tris (3,5-dimethylphenylcarbamate) derivatized cellulose bonded to silica gel. The separation of isomers may be attributed to dipole-dipole interactions between C=O groups of the CSP and C=O groups present in Rosuvastatin Calcium. In addition, hydrogen bonding interactions can occur between the solute (C=O and OH groups in Rosuvastatin Calcium) and the polar carbamate group on the CSP. Solutes having aromatic functionalities could provide additional stabilizing effects on the solute–CSP complex by insertion of the aromatic portion into the chiral cavity [24]. This type of stabilization effect may also exist in Rosuvastatin Calcium owing to the presence of aromaticity.

#### Selection of Diluent

The test solution was not clear in 100% 2-propanol or ethanol. With 0.1% TFA in 2-propanol or ethanol, the sample solution was clear but the sample solution was unstable at acidic pH, as the % of anti isomer was increasing. In 100% methanol, the sample solution was clear, but the broad peak shape was observed for the enantiomer and Rosuvastatin Calcium. Since dichloromethane was used in the mobile phase preparation, it was also selected along with methanol in the diluent. Methanol (4%) was added to the sample and sonicated to dissolve. Then the solution was made up to the mark with diluent containing dichloromethane and methanol in the ratio 96:4 (v/v). We observed good solubility, good peak shapes, and stability of the sample solution in this diluent.

#### Selection of Solvents in Mobile Phase

The sample spiked with the enantiomer, lactone, anti isomer, and 5-Oxo acid was chosen for initial method development. The following organic solvents were chosen for method development screening, and the other chromatographic conditions remain the same.

**Table:**
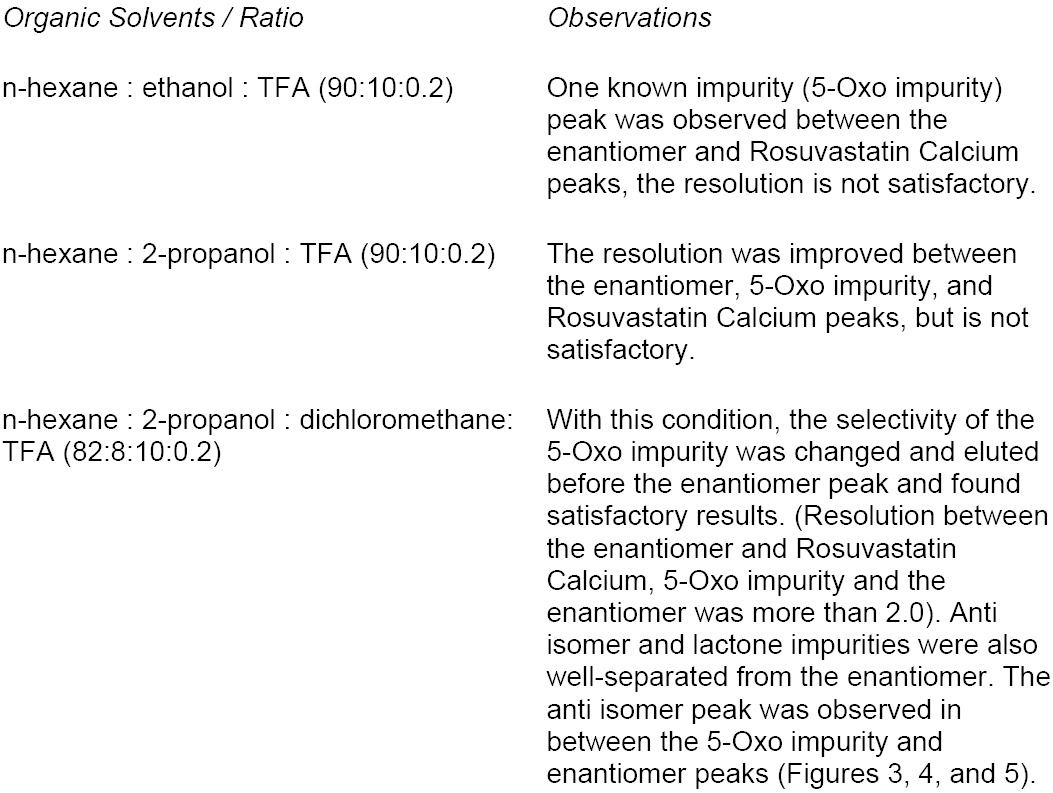


### Method Optimization by Design of Experiments

Quality-by-Design (QbD), as defined by the ICH guideline Q8 (R2), is “a systematic approach to development that begins with predefined objectives and emphasizes product and process understanding and process control, based on sound science and quality risk management.” This approach can be extended to analytical method development and should replace the obsolete trial-and-error approach in which, for example, one-factor-at-a-time (OFAT) is varied. A modern Quality-by-Design approach uses more statistical concepts with experimental design plans (also referred as Design-of-Experiments) as an efficient and fast tool for method development. The Design-of-Experiments (DoE) is defined by the ICH guideline Q8 (R2) as “a structured, organized method for determining the relationship between factors affecting a process and the output of that process” [25].

In a DoE-based QbD approach, a couple of experiments in a full or fractional factorial design are carried out, in which one or more factors are changed at the same time. Using statistic tools, the effect of each factor on the separation is calculated to define a design space, an area in which the developed method is robust. Typical examples for the use of statistic tools are the widespread use of the “Plackett-Burman” design, a highly fractionated factorial design recommended for screening experiments only, or the more advanced Box-Behnken or Central Composite designs [26].

Based on method development results, column oven temperature, 2-propanol content, and dichloromethane content in mobile phase were selected as Critical Method Parameters (CMPs). The Design–of-Experiment for the scouting and optimization runs was set up in Design-Expert 9.0.1 software by using two-level full factorial design options. The use of this statistical experimental design ensures that all important study factor effects will be expressed in the experimental data, and taken together can comprehensively explore a multifactorial design space.

USP resolutions between the enantiomer and Rosuvastatin Calcium, anti isomer, and enantiomer peaks were selected as Critical Quality Attributes (CQAs). A total of 11 runs including three runs at the center point were performed. In all of the runs, the USP resolution between the enantiomer and Rosuvastatin Calcium peaks (Response 1, Resolution-1) and USP resolution between the anti isomer and enantiomer peaks (Response 2, Resolution-2) were monitored. The results from the 11 runs are tabulated in Table 1.

**Tab. 1:**
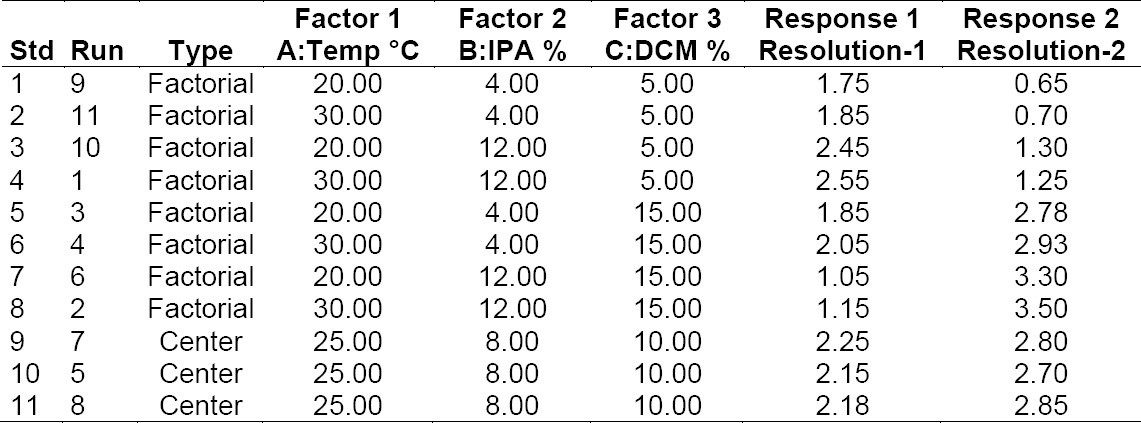
Design-of-Experiments for scouting

**Fig. 6:**
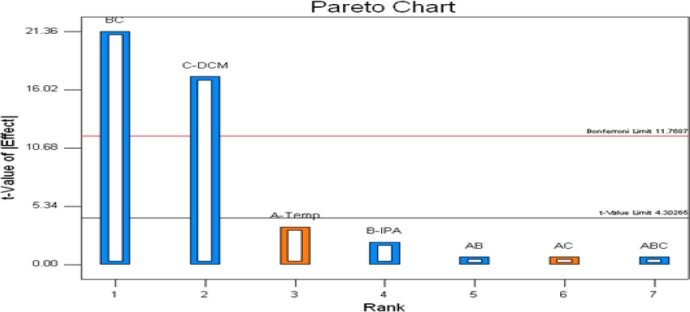
Pareto chart for the Critical Quality Attribute (CQA), USP resolution between the enantiomer and Rosuvastatin Calcium peaks (Response 1, Resolution-1)

**Fig. 7:**
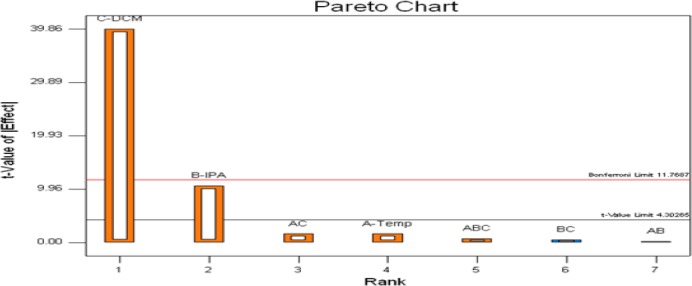
Pareto chart for the Critical Quality Attribute (CQA), USP resolution between the anti isomer and enantiomer peaks (Response 2, Resolution-2)

The effect of the Critical Method Parameters (independent variables) on the two Critical Quality Attributes (two dependent variables) was explained by using Pareto charts in Figures 6 and 7. Resolution-1 was majorly affected by C (DCM content in mobile phase) and had a mixed interaction with BC (DCM and IPA content in mobile phase). Resolution-2 was majorly affected by C (DCM content in mobile phase) only.

**Fig. 8:**
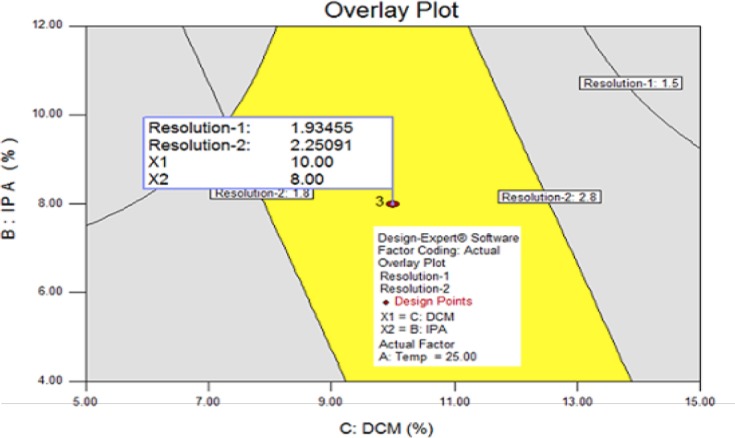
Overlay plot of Design of Experiments

The design space graph is shown in Figure 8. The definition for design space of an LC method can be a “multidimensional combination and interaction of mobile phase variables (solvent composition) and chromatographic parameters (column oven temperature) that have been demonstrated to provide assurance of results obtained with the method”. The yellow region in the design space graph indicates that the responses are in an acceptable range and the grey region shows that the responses are below the desired level [27–29]. The center point parameters (2-propanol content, 8 mL; dichloromethane content, 10 mL; and column oven temperature, 25°C) are lying in the middle of the design space, hence these parameters were finalized for normal operation.

To further verify the obtained design space, to better understand the edges of failures, and to verify the robustness of the method, two verification trials were done: one with all CMPs at significantly higher ranges and another with significantly lower ranges than the optimized condition. The obtained results were very close to the design space’s prediction and proved the built-in robustness of the method. The design space around the normal operation indicates the robustness of the method.

**Fig. 9:**
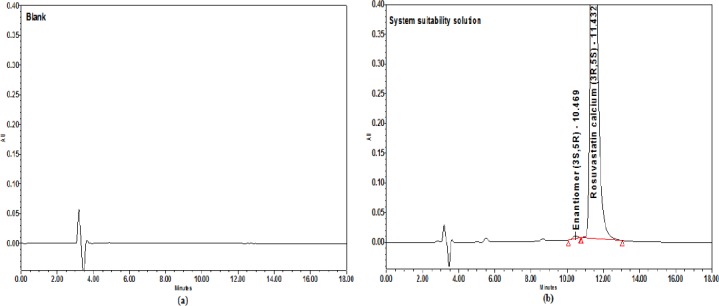
Chromatograms in the final conditions. (a) Blank (b) System suitability solution

**Fig. 10:**
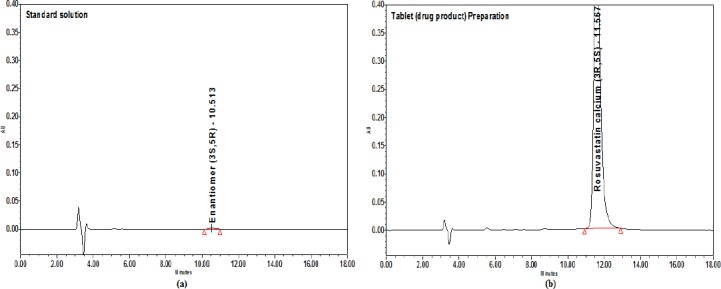
Chromatograms in the final conditions. (a) Standard solution (b) Tablet preparation

Chromatograms for the blank, system suitability solution, standard solution, and innovator tablet (Crestor) in the final chromatographic conditions are shown in Figures 9 and 10.

### Method Validation

#### Specificity/Forced Degradation Study Results

The diluent- and placebo-spiked solutions showed no peak interference with the enantiomer; moreover, the purity angle values of the enantiomer were much less than the purity threshold values, indicating the high specificity and selectivity of the method.

Each degraded sample was injected as such and spiked with the enantiomer. The peak purity for the enantiomer was ensured with a PDA detector. The enantiomer was well-separated from the obtained degradation products.

**Fig. 11:**
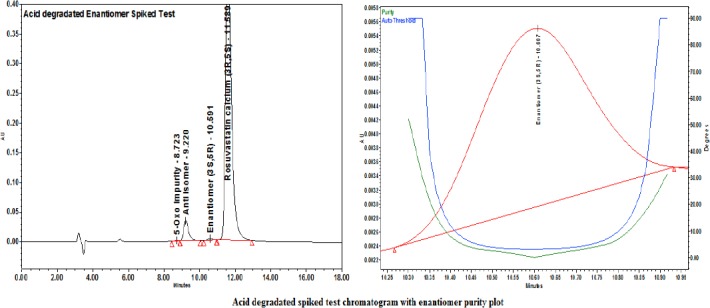
Acid degradation-spiked test with the enantiomer purity plot

**Fig. 12:**
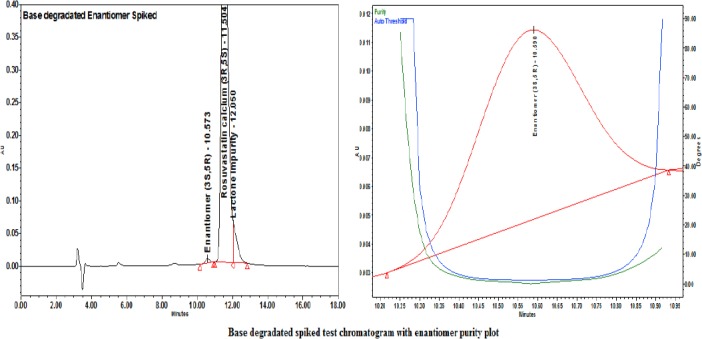
Base degradation-spiked test with the enantiomer purity plot

**Fig. 13:**
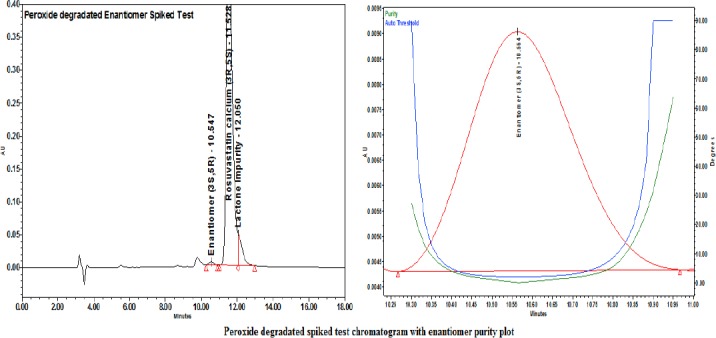
Peroxide degradation-spiked test with the enantiomer purity plot

For the degraded enantiomer-spiked test chromatograms along with the purity plots, refer to Figures 11, 12, and 13.

#### Limit of Detection and Quantification

The obtained LOD, LOQ, LOQ precision, and accuracy are given in Table 2.

**Tab. 2:**
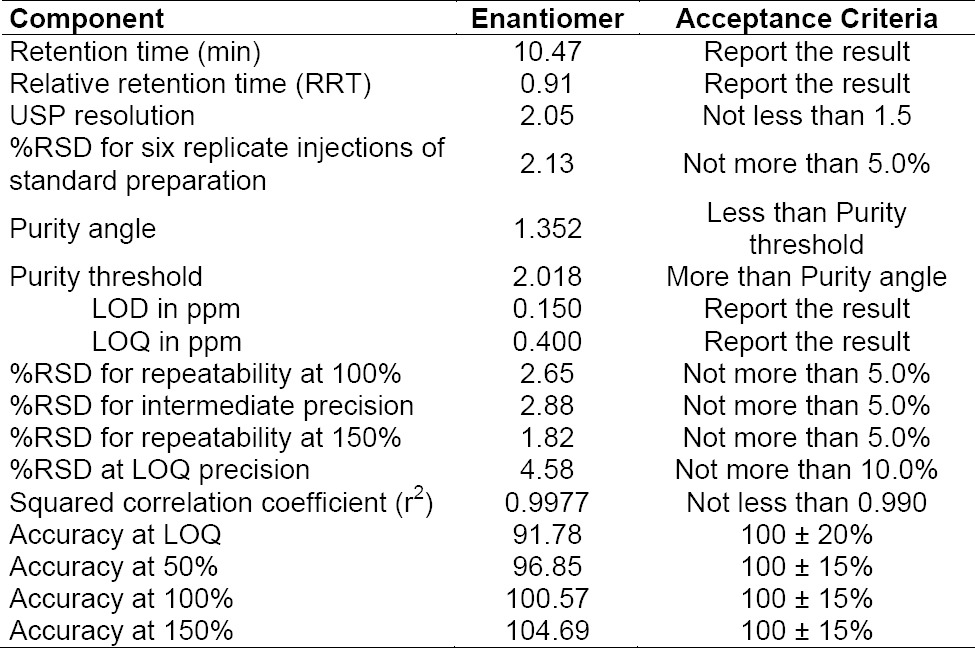
Summary of method validation for the enantiomer

#### Precision

The %RSD for the content of the enantiomer was found to be less than 5% in all of the studies. The results confirmed the high precision of the method.

#### Linearity

The calibration curve was drawn by plotting the average enantiomer peak area for triplicate injections against the concentration. The result for the squared correlation coefficient (r^2^) is shown in Table 2.

#### Accuracy

Individual and average recoveries of the three preparations and at three concentrations for the enantiomer were within 100 ± 10%.

#### Robustness

The method was more robust within the normal operating range, i.e., column oven temperature, 25 ± 5°C (factor 1); % 2-propanol, 8 ± 2% (factor 2); % dichloromethane, 10 ± 1% (factor 3); and flow rate, 1.0 ± 0.1 mL min^−1^, demonstrating the robustness of the method.

#### Solution Stability and Mobile Phase Stability

The results from the solution stability experiments confirmed that the system suitability solution and impurities-spiked test solutions were stable up to 24 hr at 10°C. The results from the mobile phase stability experiments confirmed that the system suitability solution and impurities-spiked test solutions in the mobile phase were stable up to 48 hr.

## Conclusion

A Quality-by-Design (QbD) approach to define an operating space within the design space is often based on knowledge gained through Design-of-Experiments. In this case study, we used the statistic Design-Expert 9.0.1 software to develop the simple HPLC method for quantitative estimation of the enantiomer in the drug substance and pharmaceutical dosage form. Critical method parameters like column oven temperature, 2-propanol content, and dichloromethane content in the mobile phase were successfully optimized by multivariate analysis. An operating space within the design space was established and ensured a robust HPLC method, which increased confidence in the ability to validate that method. This simple HPLC method is precise, accurate, linear, robust, and specific. Hence, it is proven that the method can be used to monitor the stability of the drug substances and drug products by quantifying the enantiomer content. The method is user-friendly and robust to operate.
